# Granulin-epithelin precursor interacts with 78-kDa glucose-regulated protein in hepatocellular carcinoma

**DOI:** 10.1186/s12885-017-3399-x

**Published:** 2017-06-10

**Authors:** Chi Wai Yip, Ching Yan Lam, Terence C. W. Poon, Tan To Cheung, Phyllis F. Y. Cheung, Sze Wai Fung, Xiao Qi Wang, Idy C.Y. Leung, Linda W. C. Ng, Chung Mau Lo, George S. W. Tsao, Siu Tim Cheung

**Affiliations:** 1 Department of Surgery, The Chinese University of Hong Kong, Hong Kong, China; 2 Department of Surgery, The University of Hong Kong, Hong Kong, China; 3 Faculty of Health Sciences, University of Macau, Macau, China; 4 Department of Anatomical and Cellular Pathology, The Chinese University of Hong Kong, Hong Kong, China; 5 School of Biomedical Sciences, The University of Hong Kong, Hong Kong, China; 6 Li Ka Shing Institute of Health Sciences, The Chinese University of Hong Kong, Hong Kong, China; 7 Department of Health, The Government of the Hong Kong Special Administrative Region, Hong Kong, China; 8 Department of Surgery, The Chinese University of Hong Kong, Prince of Wales Hospital, Shatin, Hong Kong

**Keywords:** Granulin-epithelin precursor, 78-kDa glucose-regulated protein, Hepatocellular carcinoma, Mass spectrometry, Protein interaction

## Abstract

**Background:**

Granulin-epithelin precursor (GEP) is a secretory growth factor, which has been demonstrated to control cancer growth, invasion, drug resistance and immune escape. Our previous studies and others also demonstrated its potential in targeted therapy. Comprehensive characterization of GEP partner on cancer cells are warranted. We have previously shown that GEP interacted with heparan sulfate on the surface of liver cancer cells and the interaction is crucial for GEP-mediated signaling transduction. This study aims to characterize GEP protein partner at the cell membrane with the co-immunoprecipitation and mass spectrometry approach.

**Methods:**

The membrane fraction from liver cancer model Hep3B was used for capturing binding partner with the specific monoclonal antibody against GEP. The precipitated proteins were analyzed by mass spectrometry. After identifying the GEP binding partner, this specific interaction was validated in additional liver cancer cell line HepG2 by co-immunoprecipitation using GRP78 and GEP antibodies, respectively, as the bait. GRP78 transcript levels in hepatocellular carcinoma (HCC) clinical samples (*n* = 77 pairs) were examined by real-time quantitative RT-PCR. GEP and GRP78 protein expressions were investigated by immunohistochemistry on paraffin sections.

**Results:**

We identified the GEP-binding protein as 78-kDa glucose-regulated protein (GRP78, also named heat shock 70-kDa protein 5, HSPA5). This interaction was validated in independent HCC cell lines. Increased GRP78 mRNA levels were demonstrated in liver cancer tissues compared with the paralleled liver tissues (*t*-test, *P* = 0.002). GRP78 and GEP transcript levels were significantly correlated (Spearman’s correlation, *P* = 0.001), and the proteins were also detectable in the cytoplasm of liver cancer cells by immunohistochemical staining.

**Conclusions:**

GRP78 and GEP are interacting protein partners in liver cancer cells and may play a role in GEP-mediated cancer progression in HCC.

## Background

The secretory growth factor granulin-epithelin precursor (GEP) is also known as progranulin or PC cell-derived growth factor. It constitutes of seven and half cysteine rich granulin subunits, which are known to regulate inflammation [1]. The precursor GEP protein plays different roles in a range of physiological mechanisms including neuronal survival [2, 3], fetal development [4], wound response [5] and cancer progression [6]. We previously showed that GEP was over-expressed in over 70% of human hepatocellular carcinoma (HCC) samples [7]. Functional studies showed that GEP promoted cell proliferation, invasion, chemo-resistance and immune escape in HCC [7–9].

HCC is the major form of primary liver cancers [10]. With about 745,500 deaths annually, it is the second leading cause of cancer-related death globally in 2012 [11]. Dismal prognosis have been revealed in HCC patients with less than 20% survival rates in five years [11]. This low survival rate reflects the fact that liver cancer is frequently diagnosed at an advanced stage and most patients at advanced stage of HCC could only receive systemic chemotherapies where the response rates are less than 20% [12]. Limited patients are eligible for curative therapeutic approaches which include percutaneous ablation, partial hepatectomy and transplantation [13]. However, the 5-year recurrence rate is relatively high with over 60% even after curative partial hepatectomy [14]. Although HCC occurs most frequently in Asia, incidence and mortality rates of HCC are increasing rapidly in Western countries [15] such as the United States [16]. Current technologies in HCC prognosis are unsatisfactory, hence, a thorough understanding of the mechanisms of HCC is essential for developing diagnostic approaches and for seeking alternative or supportive therapies to manage liver malignancy. GEP has been recently shown as a potential therapeutic target for HCC by using monoclonal antibody. Injection of GEP antibody can suppress the growth of HCC tumor in mouse model [17] and synergize with the anti-tumor effect of chemotherapeutic agents [18]. Therefore, a detailed understanding of the mechanisms of GEP-mediated tumorigenesis in HCC is urgently needed.

We have previously shown that heparan sulfate (HS) might act as the co-receptor, which is essential in the cell surface binding and the signaling transduction of GEP [19]. Further investigation on the primary receptor of GEP in human cancers is essential. In order to look for GEP binding partners at cell membrane, this study employed mass spectrometry to identify GEP binding proteins from membrane fraction of HCC cells. The 78-kDa glucose-regulated protein (GRP78), also referred as the heat shock 70 kDa protein 5 (HSPA5) and immunoglobulin binding protein BiP, was identified to interact with GEP. This interaction was validated by co-immunoprecipitation using GRP78 antibody as bait. Clinical analysis showed that expression of GRP78 was up-regulated in HCC tumor and correlated with GEP expression.

## Methods

### Cell lines

Two human liver cancer cell lines, Hep3B and HepG2 (HB-8064 and HB-8065, respectively, American Type Culture Collection, ATCC, Manassas, VA), were cultured in advanced minimum essential medium (AMEM) supplemented with 10% fetal bovine serum and L-glutamine at 37 °C in a 5% CO_2_ incubator. GEP-overexpressed HepG2 (HepG2-FL) and Hep3B (Hep3B-FL) were generated by lipofection of GEP plasmid as reported previously [7] and were maintained in 0.4 and 0.2 mg/ml G418 respectively in complete medium. Selection by drug was discontinued during assays. Both cell lines were characterized by the company.

### Clinical samples

HCC patients were recruited between October 2002 and September 2005 with written inform consent at Queen Mary Hospital in Hong Kong. Real-time RT-PCR expression assays were performed with the snap frozen HCC and the adjacent non-tumor (NT) tissue specimens. The tissues were formalin-fixed and paraffin-embedded for immunohistochemistry.

### Recombinant GEP binding assay

Purification of recombinant GEP (rGEP) and the binding assay were described previously [19]. Briefly, HCC cells were detached by 5 mM EDTA and were incubated with purified His-tagged rGEP. Flow cytometry was used to measure the binding of rGEP by FITC-conjugated anti-His antibody. For the determination of the non-HS binding, rGEP bound-cells were incubated with 10 μg/ml heparin for 20 min at 4 °C with vortex. Cells with residual rGEP on the cell surface were incubated with anti-His antibody for measurement of non-HS binding by flow cytometry.

### Membrane fraction enrichment

The GEP-overexpressing-Hep3B cells, Hep3B-FL, were cultured for 3 days and were then washed with ice-cold PBS buffer with 0.1% glucose and Tris-buffered sucrose solution (250 mM sucrose, 0.1% glucose, 10 mM Tris, pH 7.0). The cells were harvested by scraping and collected by centrifugation for 5 min at 500×g at 4 °C. After washing with Tris-buffered sucrose solution, cells were resuspended and homogenized in solution A (20 mM Tris, 5 mM MgCl_2_, 5 mM N-ethylmaleimide, 10 mM EDTA, complete protease inhibitor cocktail, pH 7.2). Cells debris was removed by centrifugation at 3000×g for 10 min. The supernatant containing the cellular protein complexes and the homogenized membrane-anchored protein complexes was collected. Supernatant was then centrifuged at 20000×g for an hour to collect the pellet, which contained the crude membrane fraction. The pellet was lysed in lysis buffer (20 mM Tris, 5 mM MgCl_2_, 5 mM N-ethylmaleimide, 10 mM EDTA, complete protease inhibitor cocktail, 1% Triton X-100, 20% glycerol, pH 7.2) under rotation at 4 °C for 1 h. Dissolved membrane proteins were collected from the supernatant after centrifugation at 20000×g for an hour. Membrane protein concentration was analyzed by DC Protein Assay reagents (BioRad, Philadelphia, PA).

### Co-immunoprecipitation

Co-immunoprecipitation was performed as described previously [20] with minor modifications. Briefly, cells were cultured in T175 flasks for 3 days under normal conditions until the cells reached around 80% confluence. The membrane fraction enriched protein lysate described in the earlier section was incubated with monoclonal antibody anti-GEP antibody A23 (Versitech) [17] or anti-GRP78 antibody (Cell Signaling), respectively, at a ratio of 400 μg to 2 μg. This mixture was incubated at 4 °C overnight under rotation. Antibody alone and protein lysate alone, respectively, were performed as independent control reactions and served as references for the non-specific bindings with Protein G Sepharose. For each reaction, 100 μl of equilibrated protein G-Sepharose beads (Amersham Biosciences, Piscataway, NJ) were added to the antibody-lysate mixture and incubated at 4 °C with rotation for an hour. The complexes were briefly centrifuged after incubation. The supernatant was discarded and the beads were washed with 500 μl lysis buffer for 5 times to remove any unbound proteins. The co-immunoprecipitated proteins were eluted by adding SDS sample buffer to the protein G-Sepharose beads followed by 5 min incubation at 95 °C.

### SDS-PAGE and immunoblotting

Indicated amounts of proteins were separated in denatured condition in SDS-PAGE gel. Proteins were stained by Coomassie blue or electro-transferred onto polyvinylidene fluoride membranes. For immunoblotting, the membrane was blocked by 5% skim milk and subsequently incubated overnight at 4 °C with corresponding primary antibodies. Horseradish peroxidase-labeled secondary antibodies with enhanced chemiluminescence (GE healthcare Life Sciences) was used for detection.

### Mass spectrometry

The differential protein band at about 85 kDa was excised from the Coomassie blue-stained gel, and in-gel trypsin digestion was performed as previously described [21]. Briefly, the gel was destained and reduced, followed by alkylation and digestion. C18 ZipTips (Millipore Corp., Billerica, MA) was used to desalt the tryptic digested peptides. The desalted peptides were subjected to MALDI-TOF/TOF MS (Ultraflex-III, Bruker Daltonics, Bremen, Germany). The MS and MS/MS spectra were analyzed with the FlexAnalysis program (version 3.0, Bruker Daltonics) with default parameters. The matched peptides of the MS spectrum were searched via the MASCOT search engine for the protein identity using the peptide mass fingerprinting (PMF) approach and the MS/MS ion search approach. One missed cleavage in trypsin digestion was allowed among the search parameters. Phosphorylation of serine/threonine/tyrosine, methionine partial oxidation and iodoacetamide modification of cysteine residues were selected. The error tolerance values were 50 ppm and 0.1 Da, respectively, for the parent peptides and MS/MS ion masses.

### Real-time quantitative PCR

Real-time quantitative RT-PCR was performed as described previously [7]. GRP78 primers and probe were pre-made reagents (Pre-designed TaqMan Gene Expression Assay, Life Technologies). Reagents for control 18S were Pre-designed TaqMan Assay Reagents (Life Technologies). The GRP78 mRNA expressions were examined in HCC and the corresponding non-tumor tissues (*n* = 77 pairs). The GRP78 relative levels had been normalized with control 18S and calibrator for RNA quantity and plate-to-plate variation. The mRNA expression of GEP has been determined previously in the same cohort [19] and was used for correlation analysis in this study.

### Immunohistochemistry

GEP and GRP78 proteins were examined by immunohistochemistry [7, 17]. Monoclonal anti-GEP antibody (Versitech) [17] at a dilution of 1:500 and polyclonal anti-GRP78 antibody (Cell Signaling) at a dilution of 1:250 was used in the staining. Immunohistochemical staining was performed with the Dako Envision Plus System (Dako, Carpinteria, CA). Briefly, sections were subjected to deparaffinization and hydration, then 10 min boiling in citrate buffer (pH 6) for antigen retrieval. Inactivation of endogenous peroxidase, followed by primary antibody incubation at room temperature, and signal detection by horseradish peroxidase-conjugated secondary antibody incubation at room temperature. Diaminobenzidine served as the chromogen and visualized as brown stain with hematoxylin counterstained.

### Statistical analysis

Continuous variables were assessed by Spearman’s correlation, comparison between groups by Student’s *t*-test or one-way analysis of variance as appropriate and described in the text. The Youden index (sensitivity + specificity - 1) was used to determine the optimal cutoff of GRP78 expression for prediction of survival outcome. Youden index was employed to maximize the sensitivity (true-positive fraction) and specificity (1 - false-positive fraction) of the prediction simultaneously. Descriptive parameters were analyzed by chi-squared test with Bonferroni correction. *P* values less than 0.05 were considered significant. Statistical analyses were performed by SPSS (IBM SPSS Statistics for Windows, Version 21; Armonk, NY).

## Results

### Non-HS binding sites on surface of HCC cells

**Fig. 1:**
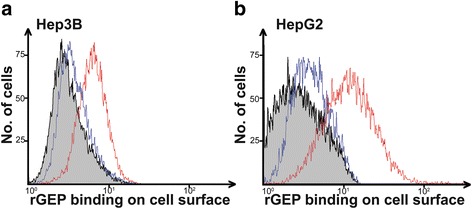
Binding of rGEP on the surface of HCC cells includes a fraction of non-HS binding. After EDTA detachment, HCC cells (**a**) Hep3B and (**b**) HepG2 were incubated with rGEP for cell surface binding. The HS-rGEP interaction was displaced by heparin. Residual binding (*blue line*) of rGEP and control binding (*red line*) of rGEP were detected by anti-His antibody and quantified by flow cytometry. *Grey* area represents the background fluorescent signal of cells without rGEP incubation. Representative histograms from three independent replicates are shown

The rGEP bound on the surface of HCC cells through HS [19] could be displaced by adding heparin. However, not all the rGEP was displaced and residual rGEP could be detected on the surface of HCC cells after heparin incubation (Fig. 1). This result suggested there were other interactions with the rGEP on the cell surface in addition to HS. The current result corroborated a previous study that there were two types of binding sites for GEP on epithelial and fibroblastic cells [22].

### Identification of GRP78 as binding partner of GEP in membrane fraction of HCC cells

Co-immunoprecipitation (co-IP) of the lysate of the membrane fraction Hep3B-FLwith GEP antibody, compared with the controls lysate alone and antibody alone respectively, only one single extra band was observed in the co-IP experiment. The SDS-PAGE gel was stained by Coomassie blue and the protein band was excised for mass spectrometry analyzes. This unknown protein was identified as GRP78 according to the masses of the trypsinized peptides in two independent runs (Table 1). Both independent runs identified the unknown protein as GRP78 (*P* < 0.05). The details of the trypsinized peptides were listed in Table 1.

**Table 1:** Summary of the peptide masses and search results of the GEP predominant interacting partner GRP78 in the liver cancer cell membrane fraction

Observed Da	Mr(expt)^a^	Mr(calc)^b^	Ppm^c^	Start-End	Miss^d^	Ions^e^	Peptide sequence
1528.6	1527.6	1527.7	−84.1	325–336	1	29	R.AKFEELNMDLFR.S + Oxidation(M)
1566.6	1565.6	1565.8	−85.4	61–74	0	19	R.ITPSYVAFTPEGER.L
1815.8	1814.8	1815.0	−84.8	198–214	1	55	R.IINEPTAAAIAYGLDKR.E
1833.7	1832.7	1832.9	−88.6	82–97	1	42	K.NQLTSNPENTVFDAKR.L
1887.8	1886.8	1887.0	−89.6	165–181	0	92	K.VTHAVVTVPAYFNDAQR.Q

^a^Experimental molecular mass (Dalton) of the peptide

^b^Relative molecular mass (Dalton) calculated from the matched peptide sequence

^c^parts per million, showing difference between the experimental and calculated masses

^d^number of missed cleavage sites

^e^ions score

**Fig. 2:**
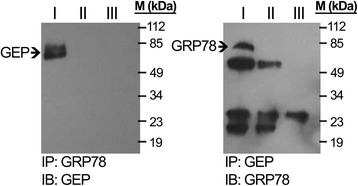
Co-immunoprecipitation using GRP78 antibody and GEP antibody respectively. Two sets of co-IP were performed using GRP78 antibody and GEP antibody, respectively, as baits. Each set of experiment contains lysate with specific antibody (I), antibody alone (II) and lysate alone (III). The proteins absorbed in the protein G beads were extracted by boiling in SDS sampling buffer and were loaded in each lane. Immunoblotting (IB) targeting GEP and GRP78 were performed. Representative blots from three independent experiments are shown

Then, membrane fraction of another HCC cells, HepG2-FL, was extracted for co-IP analysis to validate the interaction between GEP and GRP78. As show in the immunoblot of Fig. 2, using GEP antibody as bait could co-immunoprecipitate GRP78, while GRP78 antibody could immunoprecipitate GEP.

### Over-expression of GRP78 protein in HCC clinical samples

**Fig. 3:**
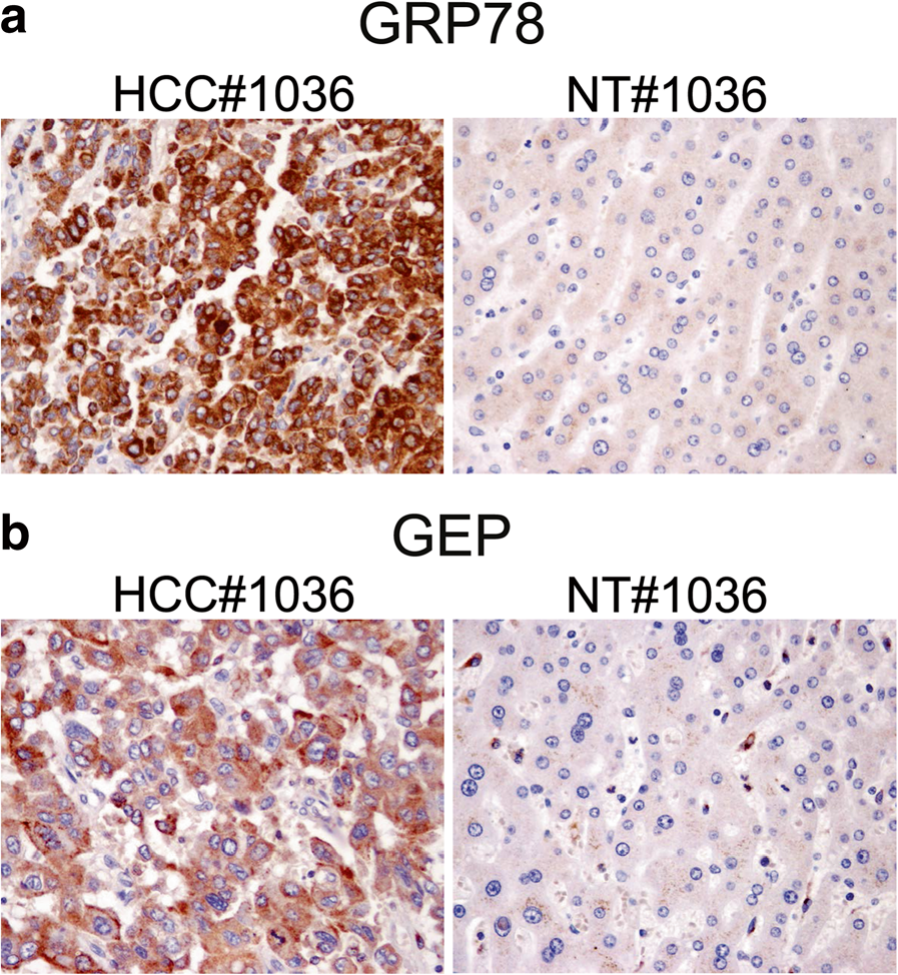
GRP78 and GEP protein expression in clinical samples by immunohistochemical staining. HCC tissue and non-tumor (NT) liver tissue from patients were formalin-fixed and paraffin-embedded. Sections were deparaffinised and hydrated. Specific antibodies to (**a**) GRP78 and (**b**) GEP were added and detected by HRP-conjugated second antibody. The sections were counterstained with hematoxylin and imaged at 400× magnification. Representative images from three individual clinical sample pairs are shown

After identifying the GRP78 as a binding partner of GEP, the protein expression of GRP78 was investigated in the HCC clinical samples by immunohistochemistry (IHC). Clinical samples that have shown over-expression of GEP in our previous studies were further examined for GRP78 by IHC. Coincident to a previous finding in HCC [23], the protein expression of GRP78 in HCC was shown to be higher than the adjacent non-tumor liver tissue (Fig. 3).

### Transcript expression of GRP78 correlates with GEP in HCC clinical samples

**Fig. 4:**
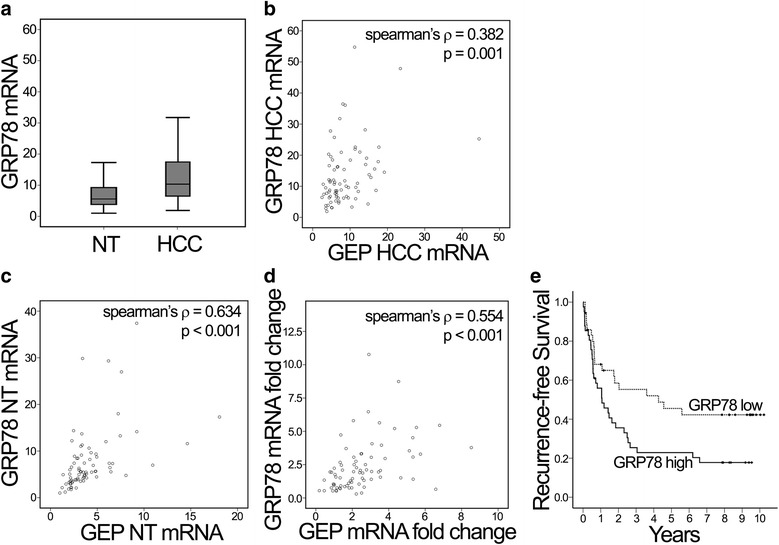
Transcript levels of GRP78 and GEP in HCC samples. **a** GRP78 expression was up-regulated in HCC with reference to their corresponding non-tumor (NT) (t-test, *P* = 0.002). (**b**-**d**) GRP78 and GEP transcript levels were significantly correlated when compared their HCC mRNA, NT mRNA and the HCC versus NT fold ratio. **e** Kaplan-Meier analysis on recurrence-free survival. Patients were segregated based on their GRP78 mRNA levels (log rank test, *P* = 0.022). The cut-off value of GRP78 expression level was determined by the Youden index

The transcript level of GRP78 was determined by QPCR from the cDNA reverse-transcripted from mRNA of patients’ HCC tumor tissues and non-tumor liver tissues. From the 77 pairs of patient samples, statistical analyses showed up-regulation of GRP78 in the HCC samples when compared to the adjacent liver tissue (*P* = 0.002) (Fig. 4a). Moreover, transcript expression levels of GEP and GRP78 correlate significantly in HCC (Spearman’s ρ correlation coefficient = 0.382, *P* = 0.001), non-tumor (Spearman’s ρ correlation coefficient = 0.634, *P* < 0.001), and the tumor-to-non-tumor fold change (Spearman’s ρ correlation coefficient = 0.554, *P* < 0.001) (Fig. 4b-d).

### GRP78 expression associates with HCC aggressive features

The mRNA expression levels of GRP78 from the HCC tumor of 77 patients were divided into two groups with high and low expression according to the cut-off defined by receiver operating characteristic (ROC) curve analysis. Clinico-pathological features were analyzed against the expression level of GRP78 (Table 2). Among them, venous infiltration showed significant correlation with high GRP78 expression. Coincidently, overexpression of GRP78 in HCC has also been reported previously to associate significantly with venous infiltration [24]. In addition, the current study demonstrated that high level of GRP78 expression correlated with poor recurrence-free survival of the HCC patients (*P* = 0.022) (Fig. 4e). Venous infiltration has been regarded as micro-metastasis and may contribute to the poor recurrence-free survival outcome of the HCC patients in GRP78 high expression group.

**Table 2:** Clinico-pathological features of GRP78 expression in HCC samples

	GRP78		
Clinico-pathological parameters	Low	High	*P* value^a^
Age			
Young (≤60)	25	29	1.000
Elderly (>60)	11	12	
Sex			
Male	30	32	0.983
Female	6	9	
Venous infiltration			
Absent	23	14	0.044^*^
Present	13	27	
Tumor stage			
Early stages	23	28	0.997
Late stages	13	13	
Tumor size			
Small (≤5 cm)	9	9	0.999
Large (>5 cm)	27	32	

^a^ *P* value by chi-squared test with Bonferroni correction

### Thapsigargin and tunicamycin supports the translocation of GRP78 to cell surface

**Fig. 5:**
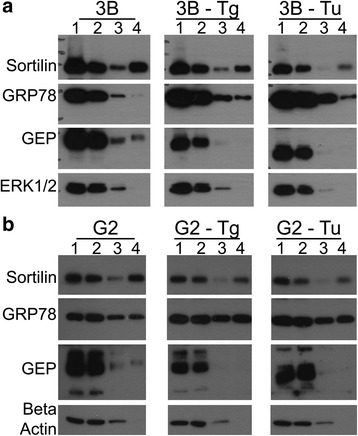
Biotinylation of cell surface proteins reflects the localization of GRP78 and GEP under thapsigargin/tunicamycin treatments in (**a**) Hep3B and (**b**) HepG2. Sortilin serves as positive control for cell surface localization; while ERK1/2 and β-actin are negative controls. 1, before loading to avidin column; 2, flow through from the column; 3, wash from the column; 4, elution of the biotinylated cell surface proteins. Starting materials is 1.5 mg/ml. Loading volume of 1 & 2 are 10 μl. Loading volume of 3 & 4 are 20 μl. Tg, 300 nM thapsigargin; Tu, 1.5 μg/ml tunicamycin. Representative blots from three independent experiments are shown

Re-localization of GRP78 to the cell surface has been reported previously. We tried to determine the re-localization in GEP expression and cell surface binding of GEP. Thapsigargin and tunicamycin, which induce ER stress by inhibiting the fusion of autophagosomes with lysosomes and inhibiting glycosylation, respectively, have been shown to induce re-localization of GRP78 [25]. In both Hep3B and HepG2 cell lines, incubation with indicated amount of thapsigargin and tunicamycin for 16 h have led to the increased overall and cell surface expression of GRP78 (Fig. 5). However, both treatments have not increased the overall and cell surface expression levels of GEP (Fig. 5).

## Discussion

In this study, we used co-IP and mass spectrometry to identify GEP binding partner from the membrane-enriched protein fraction of HCC cells. We have identified GRP78 as a binding partner of GEP in Hep3B (Table 1) and validated this interaction in another HCC cell line HepG2 (Fig. 2). GRP78 has been shown to present multifaceted subcellular positions and plays different physiological roles in different subcellular locations. Most GRP78 is retained in the ER, where it regulates unfolded protein response (UPR) by releasing ER stress transducers, PRKR-like ER kinase (PERK), inositol-requiring enzyme 1 (IRE1) and activating transcription factor 6 (ATF6), when unfolded proteins accumulate [26]. GRP78 is redistributed to mitochondria upon ER-stress, where it interacts with Raf-1 to maintain the mitochondrial permeability and inhibit ER-stress-induced apoptosis [27]. Accumulating evidence demonstrated the re-localization of GRP78 to plasma membrane, especially in the cancer cells that are under stress [28]. Both ER stress and therapeutic resistance promote the expression level and cell surface translocation of a subfraction of GRP78 [25, 29]. GRP78 on the cell surface has been shown to regulate signaling pathways [30–33]. Additionally, several molecules were shown to support the surface re-localization of GRP78. Mtj-1 and prostate apoptosis response 4 (PAR4) were shown to transport GRP78 towards plasma membrane respectively in murine tumor cells [34] and in prostate cancer cells [35]. In the presence of activated form of alpha2-macroglobulin (α2M), GRP78 was shown to translocate to plasma membrane in HCC cells [33].

With respect to the nature of GRP78 relocalization to cell surface, we attempted to determine if this re-localization increased endogenous GEP expression or on the cell surface of HCC cells; and if GRP78 contributed to cell surface binding of rGEP. Consistent with a previous study [25], higher level of cell surface GRP78 was observed in HCC cells under the treatment of thapsigargin and tunicamycin (Fig. 5). However, GRP78 re-localization did not affect the cell surface levels of GEP (Fig. 5).

Clinical significance of GRP78 expression has been analyzed. Coincident to a previous study of GRP78 at the protein level [23], we demonstrated over-expression of GRP78 in HCC tumor tissues compared to the non-tumor counterparts at the transcript levels. Furthermore, GRP78 over-expression was significantly associated with venous infiltration (Table 2) and poor recurrence-free survival (Fig. 4), implying an important role of GRP78 in HCC aggressiveness. Strikingly, expression levels of GEP are significantly correlated with the expression levels of GRP78 in both tumor and non-tumor tissues, indicating an association between GEP and GRP78 expression in HCC.

The functional roles of GRP78 in HCC have been revealed in previous studies. Over-expression of GRP78 and the consequential activation of UPR have been indicated to confer drug resistance on HCC against sorafenib and doxorubicin [36, 37]. GRP78 also plays a role in invasion and metastasis of HCC cells through the activation of FAK [38]. In the presence of the activated form of alpha2-macroglobulin (α2M), GRP78 was shown to translocate to plasma membrane and facilitate the interaction between c-Src and EGFR, leading to invasion and metastasis [33]. On the other hand, GEP has been shown to co-express with ABCB5 and protect HCC cells from chemotherapeutic agents including doxorubicin [8]. The expression of GEP in HCC cells also contributes to the invasiveness of HCC [7]. Therefore, the cell surface and intracellular interaction between GEP and GRP78 warrants further investigation to delineate their roles in HCC tumorigenesis.

## Conclusions

In summary, this study identified GRP78 from the membrane fraction of HCC cells as a binding partner of GEP. Their interaction may shed light on the multifaceted roles of both GRP78 and GEP in HCC tumorigenesis, especially their mechanistic relationship in cancer progression and drug resistance.

## Abbreviations

BiP: immunoglobulin binding protein
GEP: granulin-epithelin precursor
GRP78: 78-kDa glucose-regulated protein
HCC: hepatocellular carcinoma
HSPA5: heat shock 70-kDa protein 5
